# Supplementary figures and images for: Predicting early cessation of exclusive breastfeeding using machine learning techniques
Source: PLoS One. 2025 Jan 9;20(1):e0312238. doi: 10.1371/journal.pone.0312238 (PMC11717195; doi:10.1371/journal.pone.0312238)

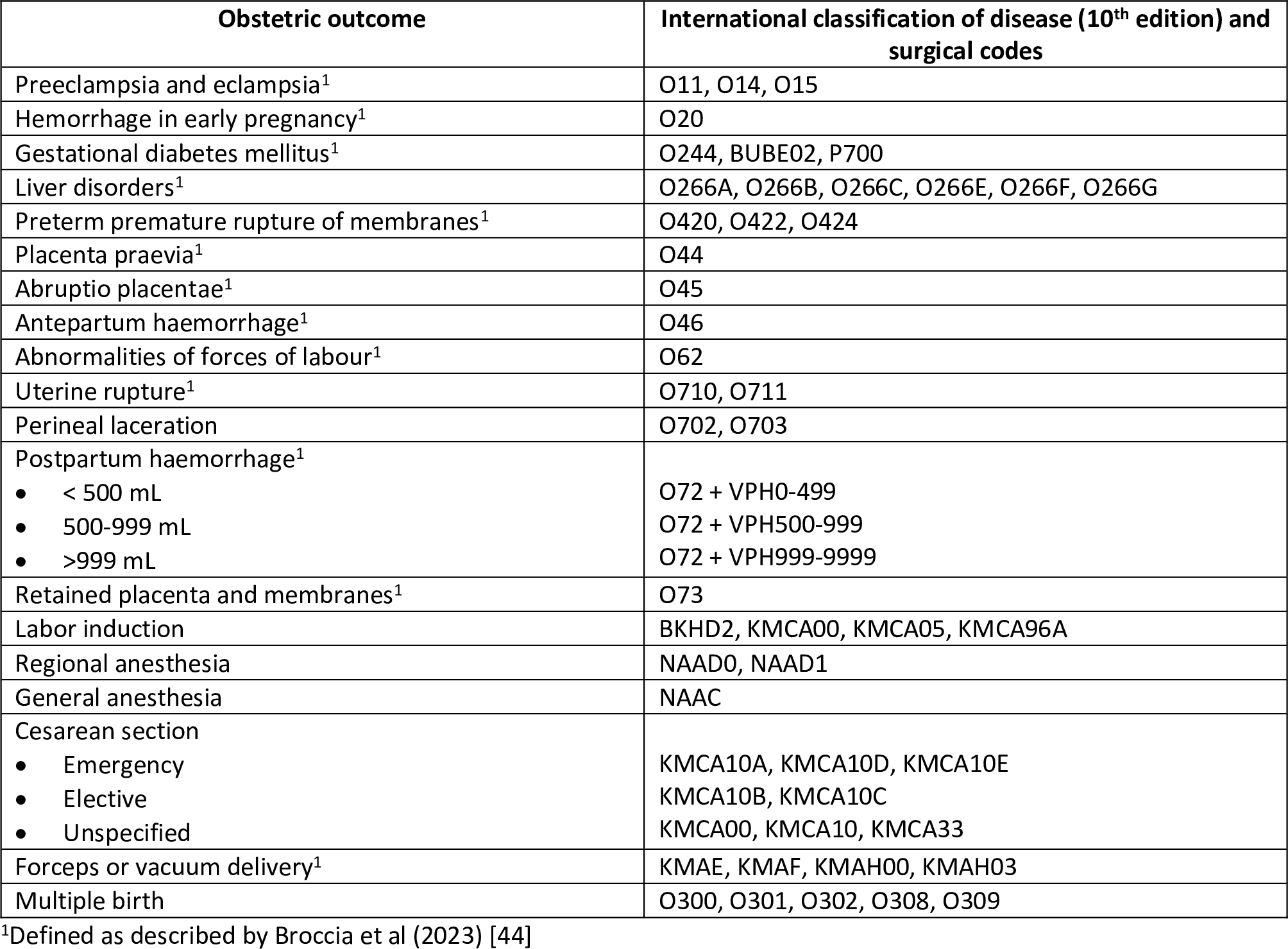

Supplement: S1 Table — (TIF) [file pone.0312238.s001.tif]

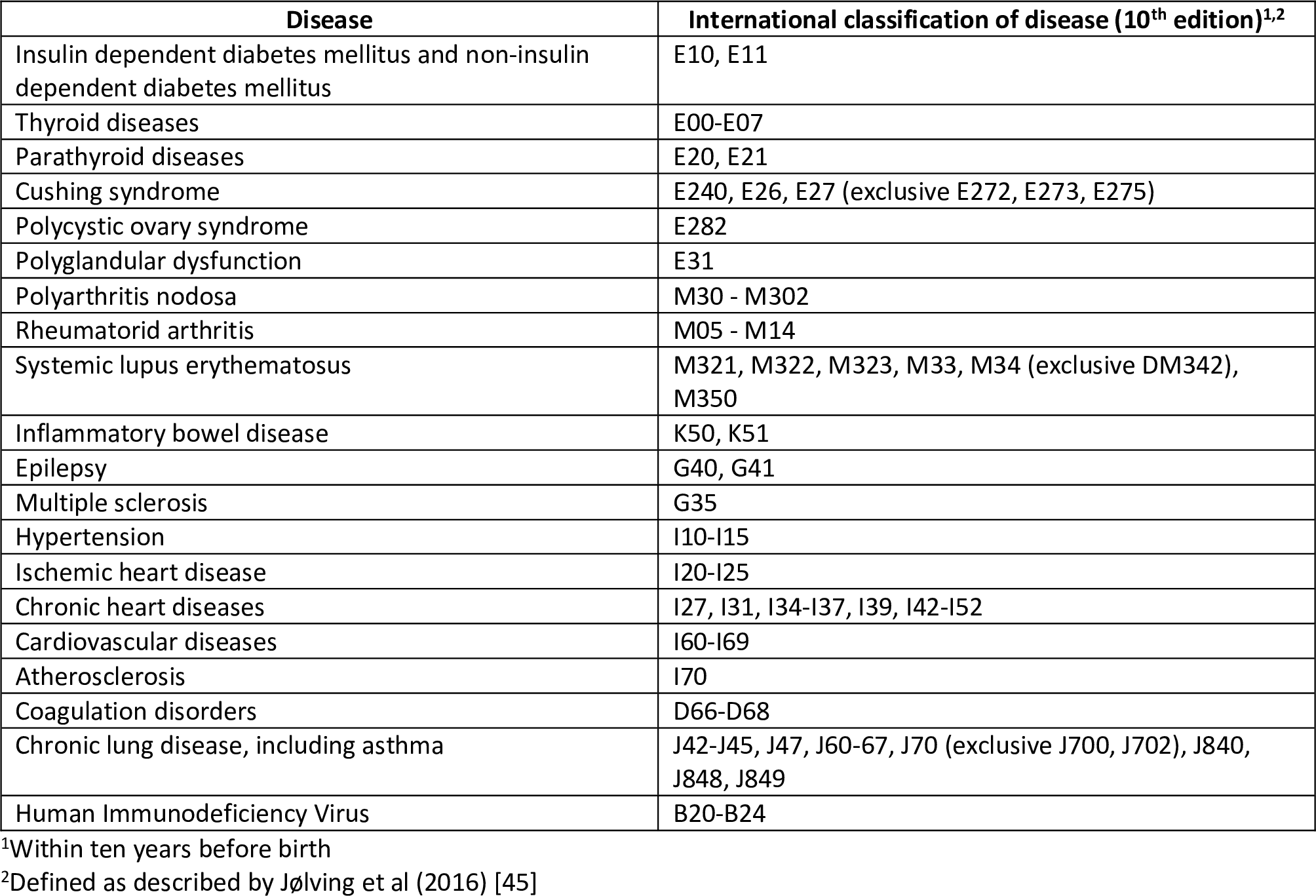

Supplement: S2 Table — (TIF) [file pone.0312238.s002.tif]

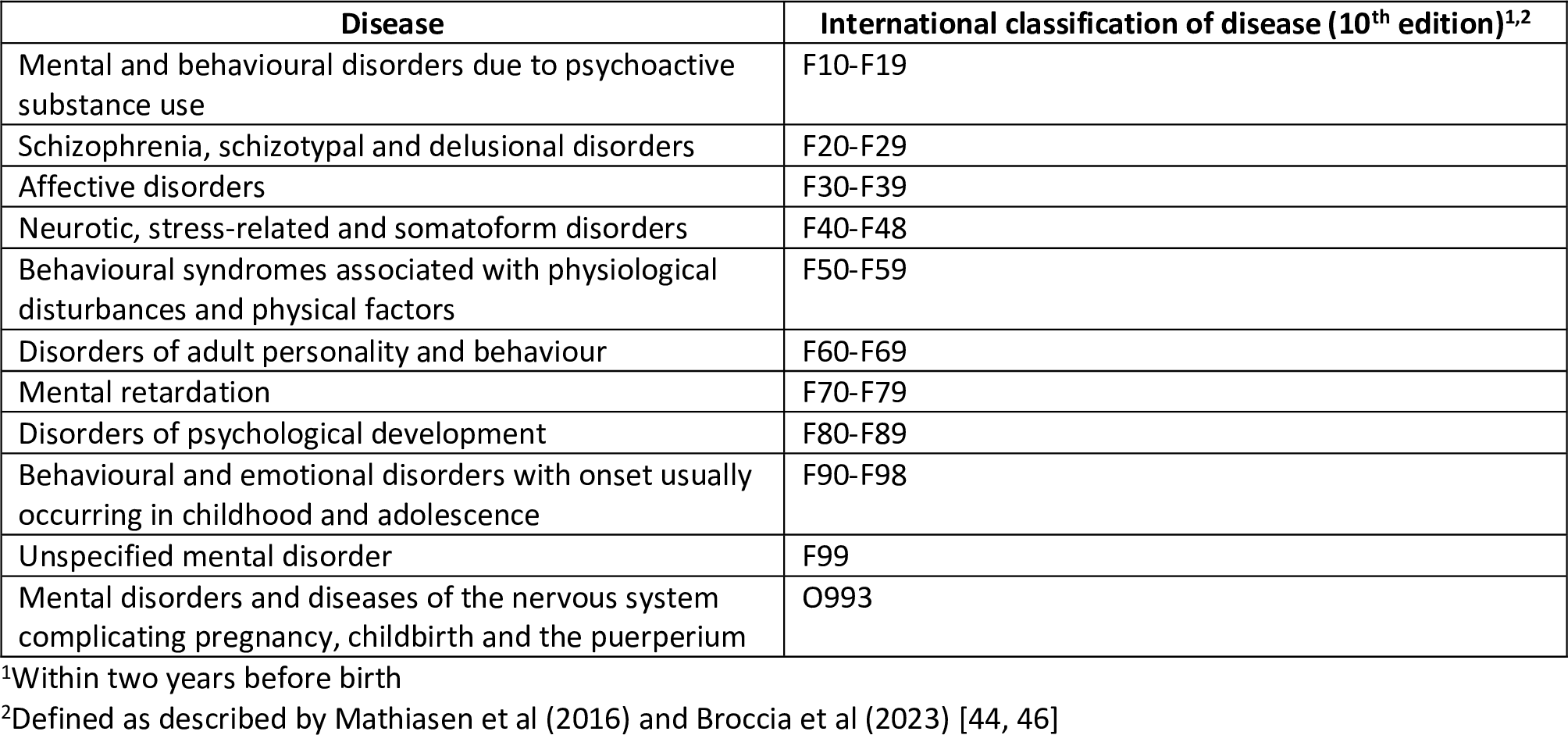

Supplement: S3 Table — (TIF) [file pone.0312238.s003.tif]

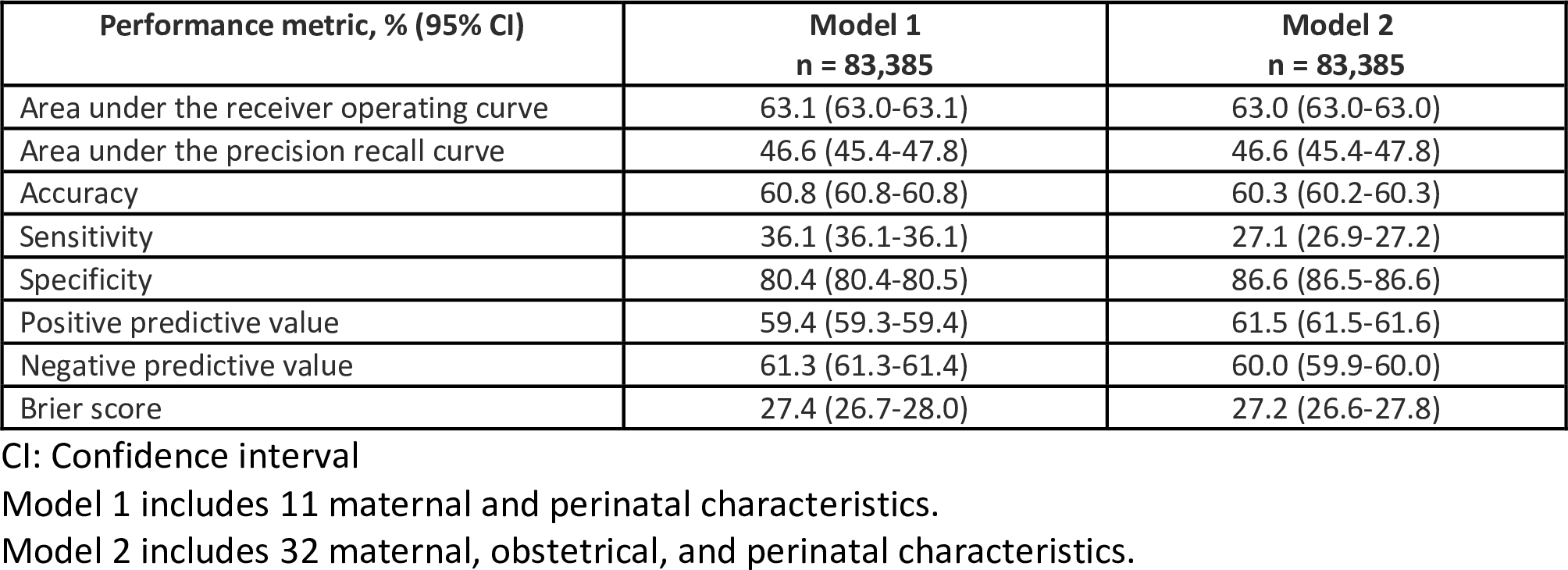

Supplement: S4 Table — (TIF) [file pone.0312238.s004.tif]

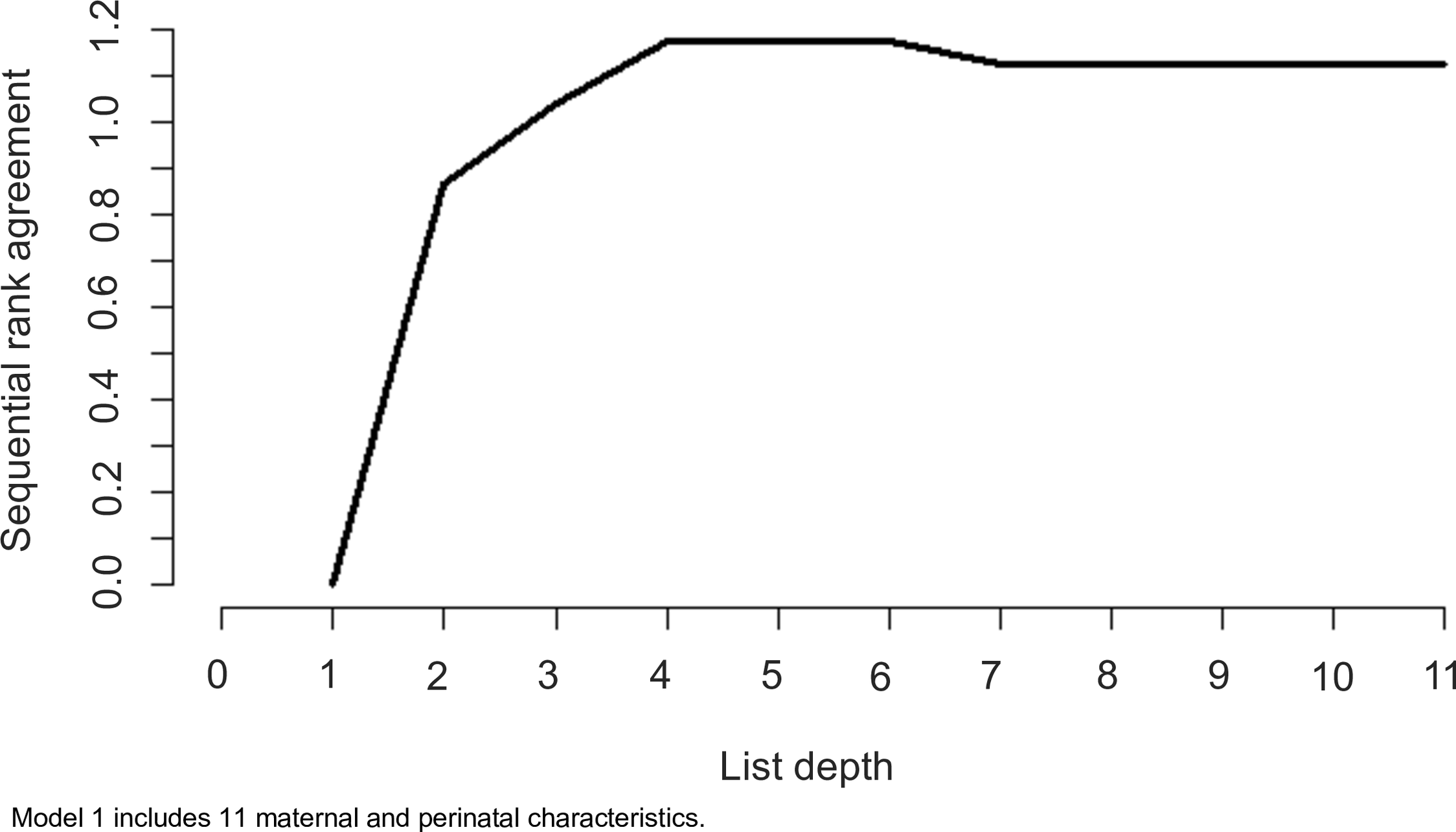

Supplement: S1 Fig — (TIF) [file pone.0312238.s005.tif]

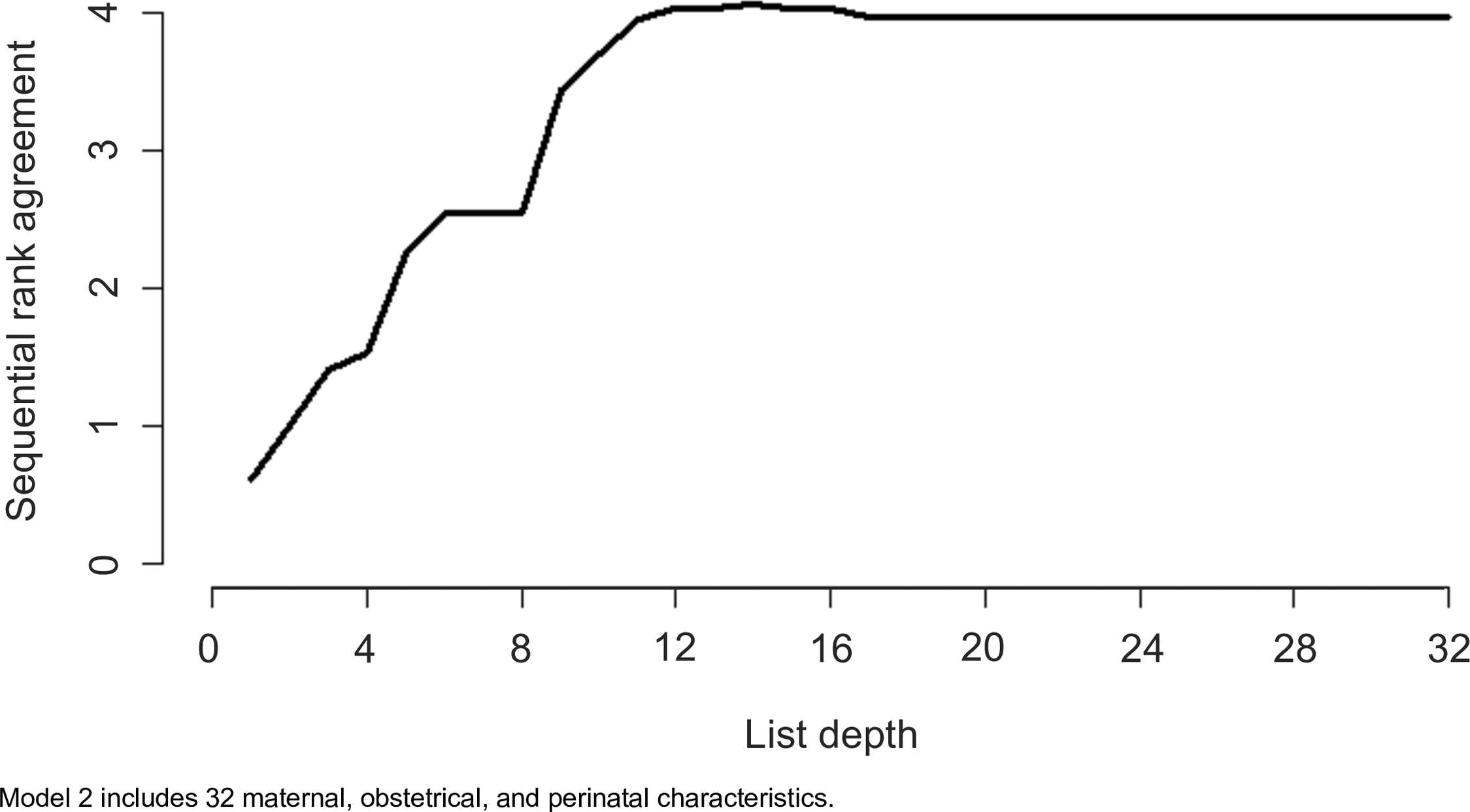

Supplement: S2 Fig — (TIF) [file pone.0312238.s006.tif]
